# Simulation of Subject-Specific Progression of Knee Osteoarthritis and Comparison to Experimental Follow-up Data: Data from the Osteoarthritis Initiative

**DOI:** 10.1038/s41598-017-09013-7

**Published:** 2017-08-23

**Authors:** Mimmi K. Liukkonen, Mika E. Mononen, Olesya Klets, Jari P. Arokoski, Simo Saarakkala, Rami K. Korhonen

**Affiliations:** 1 Department of Applied Physics, University of Eastern Finland, Kuopio, Finland; 2 Research Unit of Medical Imaging, Physics and Technology, University of Oulu, Oulu, Finland; 3 Medical Research Center, University of Oulu and Oulu University Hospital, Oulu, Finland; 4 Department of Physical and Rehabilitation Medicine, Helsinki University Hospital, Helsinki, Finland; 5 University of Helsinki, Helsinki, Finland; 6 Department of Diagnostic Radiology, Oulu University Hospital, Oulu, Finland; 7 Diagnostic Imaging Centre, Kuopio University Hospital, Kuopio, Finland

## Abstract

Economic costs of osteoarthritis (OA) are considerable. However, there are no clinical tools to predict the progression of OA or guide patients to a correct treatment for preventing OA. We tested the ability of our cartilage degeneration algorithm to predict the subject-specific development of OA and separate groups with different OA levels. The algorithm was able to predict OA progression similarly with the experimental follow-up data and separate subjects with radiographical OA (Kellgren-Lawrence (KL) grade 2 and 3) from healthy subjects (KL0). Maximum degeneration and degenerated volumes within cartilage were significantly higher (*p* < 0.05) in OA compared to healthy subjects, KL3 group showing the highest degeneration values. Presented algorithm shows a great potential to predict subject-specific progression of knee OA and has a clinical potential by simulating the effect of interventions on the progression of OA, thus helping decision making in an attempt to delay or prevent further OA symptoms.

## Introduction

Osteoarthritis (OA) is a degenerative joint disease causing pain and limitations in joint movement. At tissue level, OA affects predominantly articular cartilage, although other tissues, such as bone, meniscus, and synovium, are also involved. Indirectly, this may lead to social isolation, decrease in work ability, depression, i.e., decrease in the general quality of life^1^. Currently in Europe, it has been approximated that nearly 5–15% of people aged 35–74 years have knee OA^2^. Increasing health issues due to knee OA lead to severe economic burden in societies. For instance, it has been estimated that the total costs of arthritis (including direct and indirect costs) are approximately 2.5% of the gross domestic product in the US^3^, which corresponds to more than USD $400 billion. Despite a large research effort worldwide, there is no cure for OA, and consequently, the best and most cost-effective treatment option at the moment would be prevention. However, there are no methods which would quantitatively predict subject-specific progression of OA. This would be necessary when planning the most suitable preventative and personalized interventions for OA treatment.

Obesity is one of the major risk factors for OA^4, 5^ and ~50% of OA can be attributed to overweight and obesity^6^. It has been shown that subjects with BMI over 30 kg/m^2^ have 6.8 times higher chance to develop knee OA compared to normal weight subjects^7^. The knee joint of an obese person experiences substantial mechanical loads that can lead to OA and degenerative changes in the composition, structure and mechanical properties of articular cartilage^8, 9^. However, at the individual level, it is currently not possible to predict whether mechanical overweight-induced overloading leads to the onset and progression of knee OA, and if it does, to what extent.

The diagnosis of OA is usually based on the clinical examination and conventional radiography. However, this traditional diagnostic approach is insensitive to the early stages of OA^10^, since radiography does not reveal early degenerative tissue changes in cartilage tissue and potential OA subjects may not feel pain in their knees. The Kellgren–Lawrence (KL) grading^11^ is the most commonly used X-ray based grading criteria for assessing the severity and progression of knee OA^12^ and making treatment decisions. KL grading focuses on joint space narrowing and osteophyte development, and therefore, it cannot directly assess changes in cartilage structure and composition. On the other hand, magnetic resonance imaging (MRI) is a non-invasive method to image soft tissues. Compared to X-ray or even computed tomography (CT), MRI is capable of detecting and measuring changes in articular cartilage morphology and composition in 3D^13–15^. However, MRI information gives only imaging insight of the current knee joint condition, but it does not provide any information of the future status of the knee.

Mechanical stress plays a significant role in OA pathogenesis^16^. Computational mechanical models have been successfully used for analyzing changes in cartilage and menisci contact mechanics^17–21^, and describing how they relate to the onset and progression of OA^22–24^. In addition to contact mechanics analyses, computational models have been investigated to study soft tissue adaptation such as collagen remodelling in different soft tissues^25–28^, cartilage growth^29–31^ and damage^32, 33^.

Even though computational modelling has been widely used for investigating knee joint function, cartilage stress levels, and soft tissue adaptation and growth, there is lack of quantitative methods for predicting subject-specific progression of knee OA. Recently, we developed a novel cartilage degeneration algorithm based on tissue overloading to predict the progression of OA^34^. In that study, only a general behaviour of OA progression of two subject groups (normal weight and obese) was tested with the algorithm in a population-based manner. No information was provided on the ability of the algorithm for personalized prediction of the progression of knee OA or to separate groups with different levels of OA. Therefore, the aim of the current study is to investigate the ability of the algorithm to separate clinically healthy but obese subjects, diagnosed from the baseline radiographs, into different KL grade groups based on the predicted level of cartilage degeneration. Subsequently, prediction results are compared to clinical radiography (KL grade) and MRI findings of the cartilage tissue degeneration at the 4-year follow-up.

## Results

### Osteoarthritis Initiative Subjects

**Figure 1:**
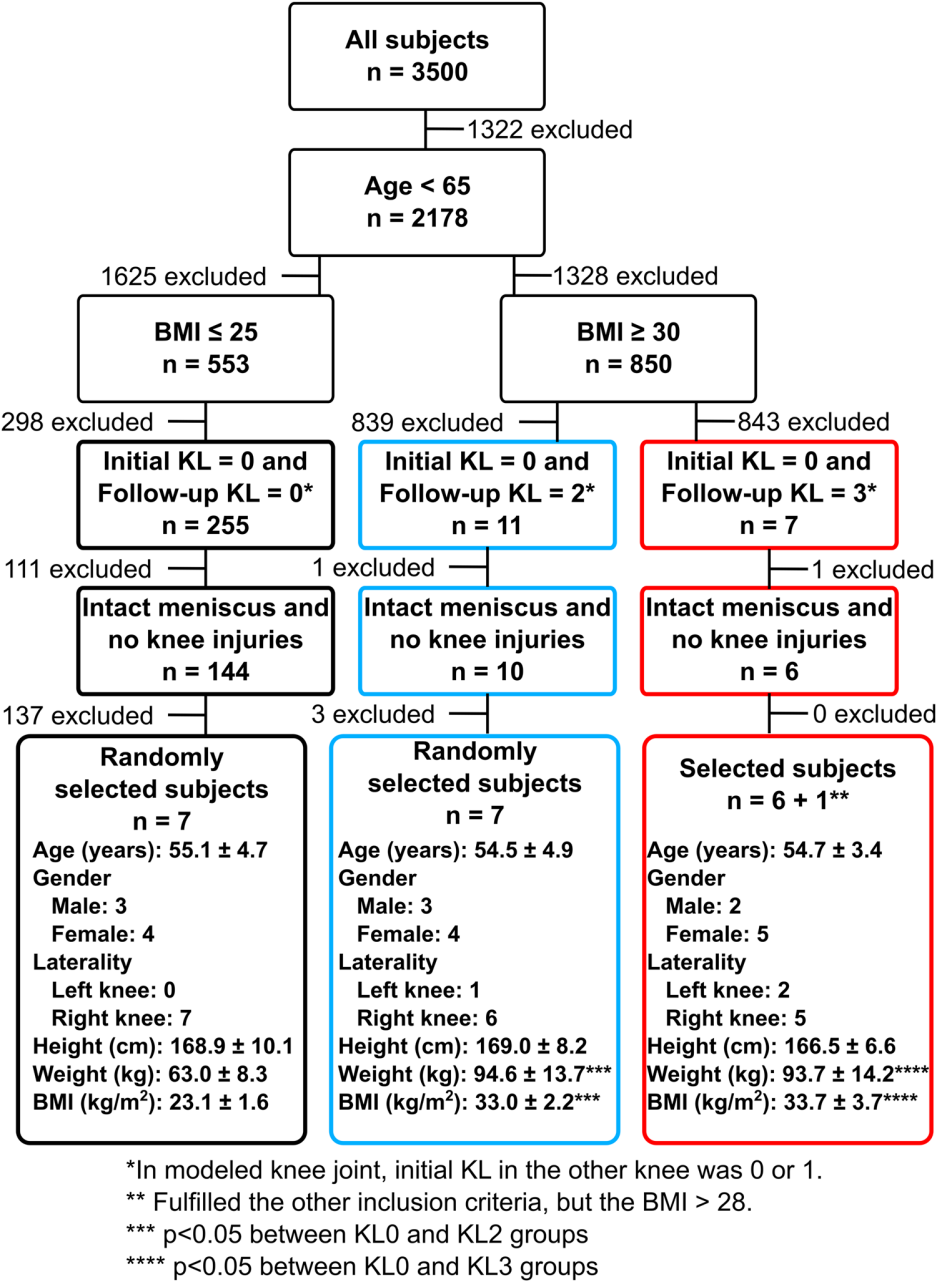
Subject characteristics (mean ± sd) and **i**nclusion criteria for selecting subjects from OAI database to three different KL groups. In KL0 group subjects’ BMI were less than 25 and in KL2 and KL3 groups more than 30. Except BMI of one KL3 subject was more than 28 since there were only 6 subjects whose BMI was more than 30 and who fulfilled all other inclusion criteria. Inclusion criteria are for knee joints used in FE modelling (3 left and 18 right), but in addition initial KL for non-modelled knee joint had to be 0 or 1.

Total of 21 subjects with radiographically intact knee joint (KL-grade = 0) at the baseline were selected from the Osteoarthritis Initiative Database (OAI–http://www.oai.ucsf.edu/) (Fig. 1). All subjects were less than 65 years old and they had not had meniscus or knee injuries (never operated meniscus or cartilage, never injured badly enough to limit the ability to walk for at least two days) (see exact inclusion criteria from Fig. 1). Subjects were divided into three different groups: KL0, KL2, and KL3, based on the 4-year follow-up KL-grades (Fig. 1). In KL0 group (N = 7, BMI = 23.1 ± 1.6 kg/m^2^), KL-grade remained unchanged after 4-year follow-up, while in KL2 (N = 7, BMI = 33.0 ± 2.2 kg/m^2^) and KL3 (N = 7, BMI = 33.7 ± 3.7 kg/m^2^) groups KL-grade increased from 0 to 2 or 3, respectively, during the follow-up period. Note that KL0 group represent normal weight subjects, while KL2 and KL3 represent overweight subjects. There was no statistical difference in BMI between the two latter groups (*p* > 0.05) even though their OA grade was different.

### Cartilage degeneration algorithm

**Figure 2:**
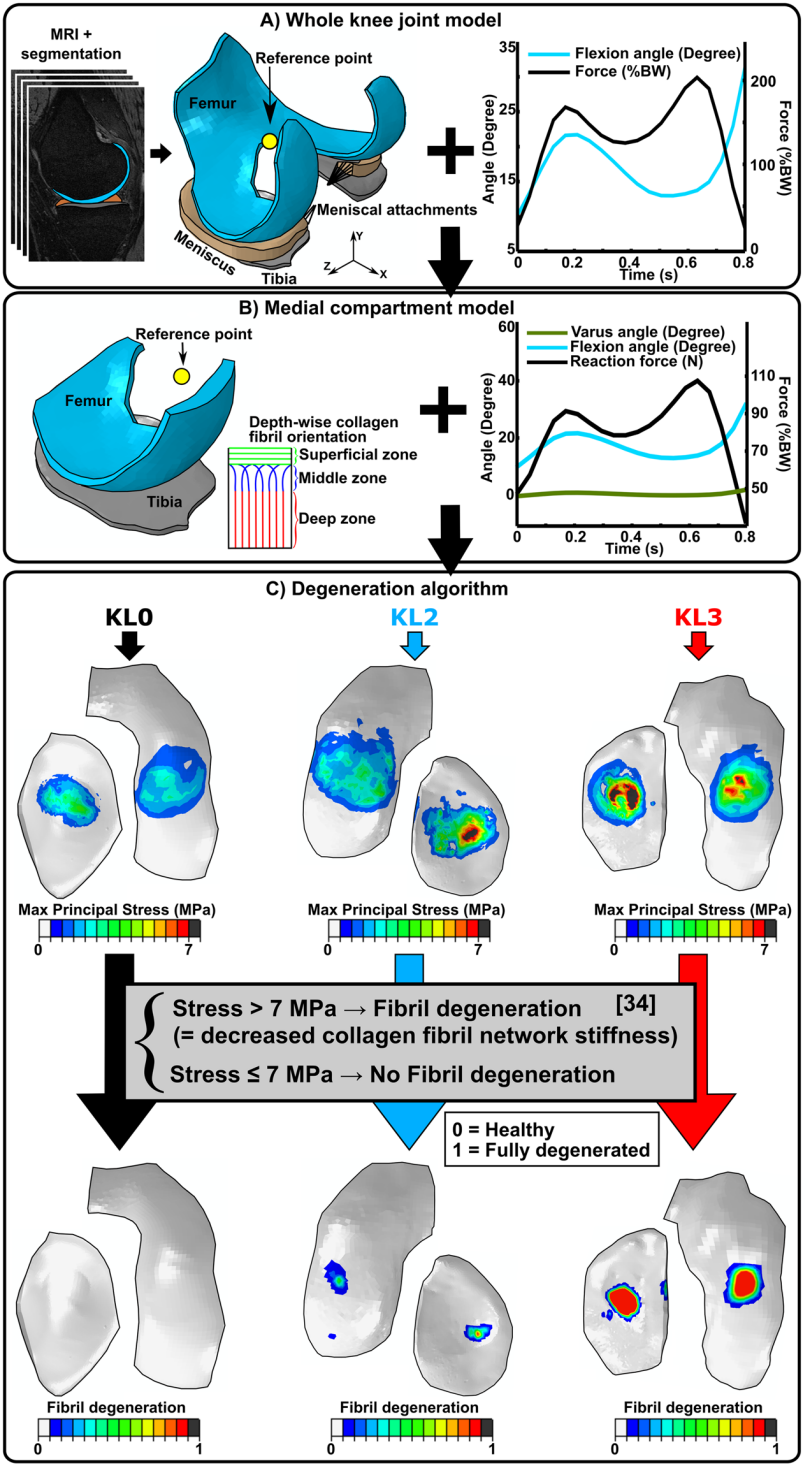
Method for predicting the level of knee osteoarthritis using cartilage degeneration algorithm. (**A**) Knee joints (*N* = 21) were manually segmented from MRI images taken from OAI database and 3D geometries were created. Gait obtained from literature^63, 64^ was implemented into the finite element models of the whole knee joint. (**B**) Medial compartment models were created to predict the level of medial knee OA (see justification behind that from Results section). Outcome force and rotations from the whole knee joint model were implemented into the compartment model to ensure similar knee joint motion in the compartment model as in the whole knee joint model. (**C**) Finally, cartilage degeneration algorithm was implemented into the medial compartment models to predict the level cartilage degeneration in three different KL groups (KL0, KL2 and KL3) after 100 iterations. In the algorithm, cartilage fibril degeneration occurs if tensile stress exceeds 7 MPa failure limit^34^, decreasing collagen fibril network stiffness (See more details in Method section).

**Figure 3:**
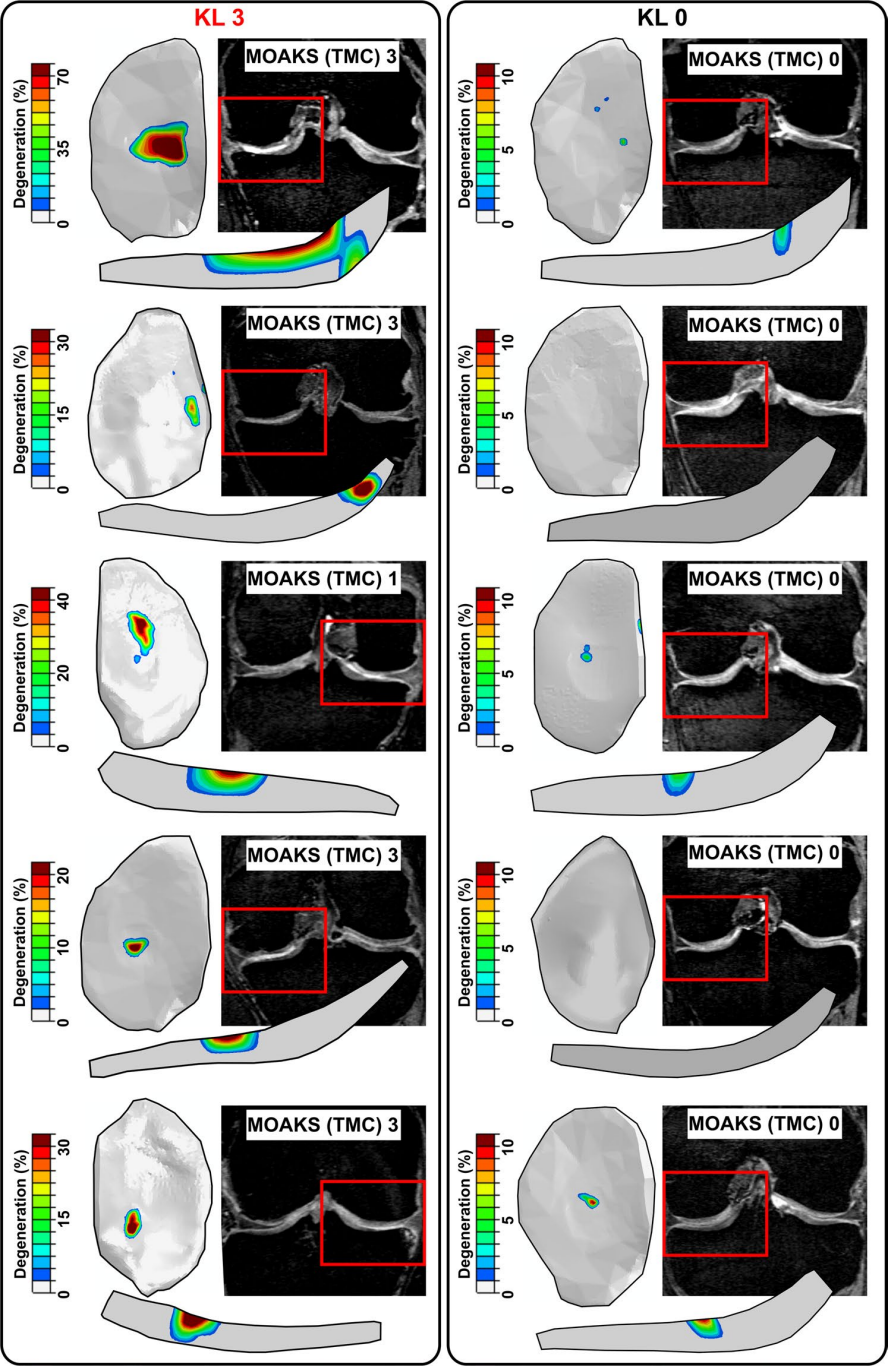
Cartilage degeneration distributions and MRI MOAKS grades for five selected KL3 and KL0 subjects. MOAKS grades are from central part of medial tibial cartilage (TMC = tibia medial central).

Cartilage degenerations were simulated for three different KL grade groups (KL0, KL2 and KL3) using the cartilage degeneration algorithm (see more from Methods)^34^. Predicted (computed) degeneration levels were compared to cartilage degenerations obtained from 4-years follow-up radiographic KL grading and MRI scoring using simplified MOAKS grading (described in Methods section). Since MOAKS grading showed that most of the cartilage degeneration occurs on the medial side of the knee, only medial compartment models were used for investigating cartilage degeneration. Workflow for degenerative simulations can be divided into three steps. First, subject-specific whole knee joint geometries were constructed from the 3D DESS MRI data based on the baseline visit (Fig. 2A). Subsequently, segmented geometries and gait cycle loading^34^, scaled to match to the subject’s body weight, were implemented into the finite element models. Finally, medial compartment models were constructed based on whole knee joint model simulations (Fig. 2B) and the collagen fibril degeneration algorithm was implemented into the models (see details in Methods section) (Fig. 2C). Predicted degenerations and MOAKS gradings from the central part of the medial tibial cartilage from five KL3 and KL0 subjects are shown in Fig. 3. In KL3 group, peak degenerations within cartilage were considerably higher and degenerated areas were larger compared to KL0 group. The same finding was verified with MOAKS grades in the medial compartment, *i*.*e*. MOAKS grades and cartilage degenerations in KL3 group were higher compared to those in KL0 group.

### Prediction of cartilage degeneration levels

**Figure 4:**
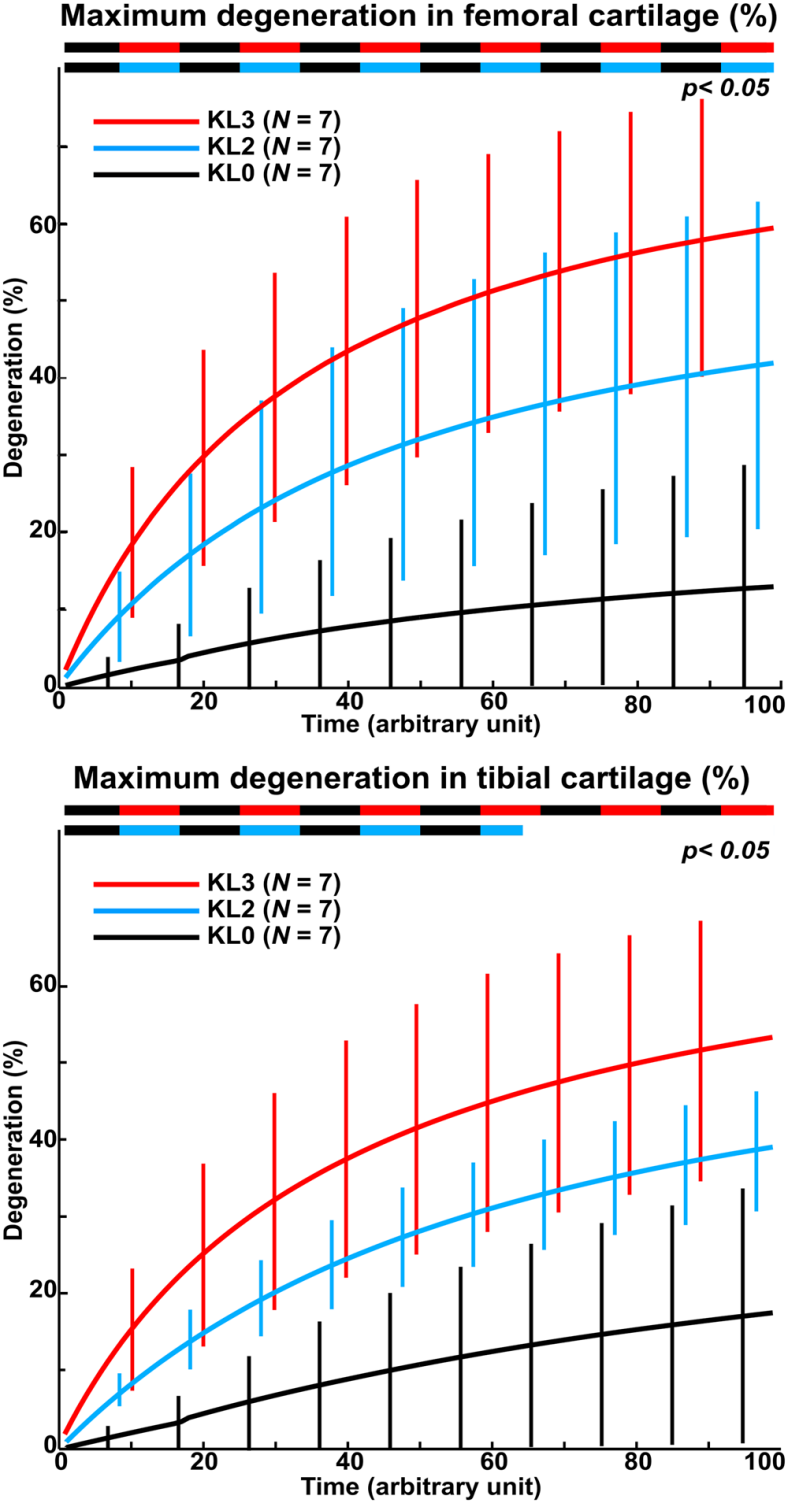
Maximum degeneration (mean ± 95% CI) as a function of time (arbitrary unit) in medial femoral (upper) and tibial (lower) cartilages. Two colored dashed lines represents significant differences between different groups.

**Figure 5:**
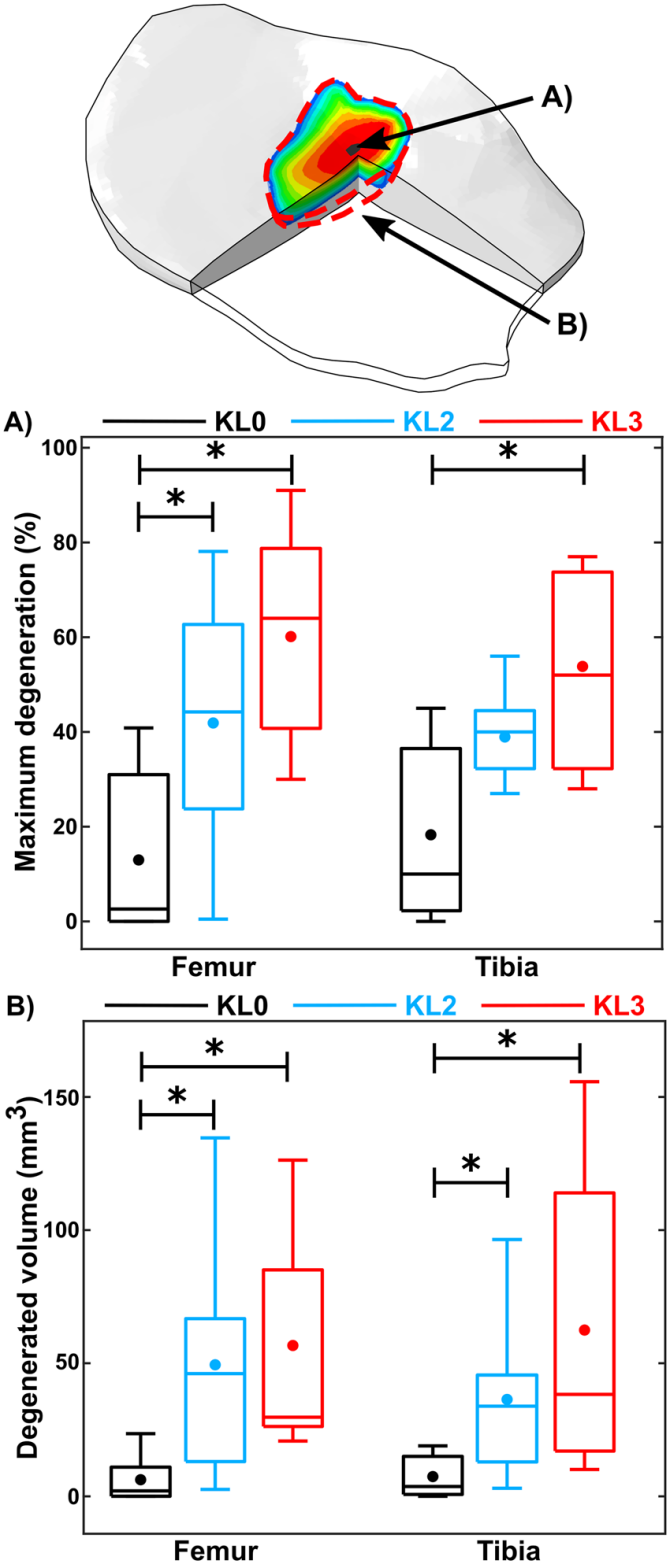
Predicted (**A**) maximum degenerations and (**B**) degenerated volumes of different KL groups in medial femoral and tibial cartilages. Circle (•) and crossline (—) represent mean and median values, respectively. *p < 0.05.

Maximum degeneration in medial femoral cartilage was significantly (*p* < 0.05) higher in KL2 and KL3 groups compared to KL0 group during all 100 iterations (Fig. 4). On the other hand, in medial tibial cartilage, maximum degeneration during all iterations was significantly (*p* < 0.05) higher in KL3 group compared to KL0 group, but differences between KL2 group compared to KL0 group were significant (*p* < 0.05) only during first 65 iterations (Fig. 4). The average of maximum degeneration in medial femoral cartilage after 100 iterations was ~two and ~four times higher in KL2 and KL3 groups, respectively, compared to KL0 group (*p* < 0.05, Fig. 5A). The difference in maximum degeneration in medial femoral cartilage between KL3 and KL2 groups was not statistically significant (*p* > 0.05), though the average of maximum degeneration was 45% higher in KL3 group. In medial tibial cartilage, statistically insignificant (*p* > 0.05) one time and significant (*p < *0.05) two times higher maximum degenerations were seen between KL0 and KL2 groups, and between KL0 and KL3 groups, respectively (Fig. 5A). The average of maximum degeneration in medial tibial cartilage was 37% higher in KL3 than KL2 group, but this difference was not significant (*p* > 0.05).

The mean volumes of degenerated elements after 100 iterations in medial femoral cartilage were ~seven and ~nine times higher in KL2 and KL3 groups, respectively, compared to KL0 group (*p* < 0.05, Fig. 5B). Moreover, mean degenerated volumes were on average 16% higher in KL3 group compared to KL2 group, but the difference was not statistically significant (*p* > 0.05). Correspondingly, in medial tibial cartilage, ~four and ~eight times higher degenerated volumes were observed in KL2 and KL3 groups compared to KL0 group (*p* < 0.05). A difference of 68% between KL2 and KL3 groups was observed, but it was not significant (*p* > 0.05) (Fig. 5B).

**Figure 6:**
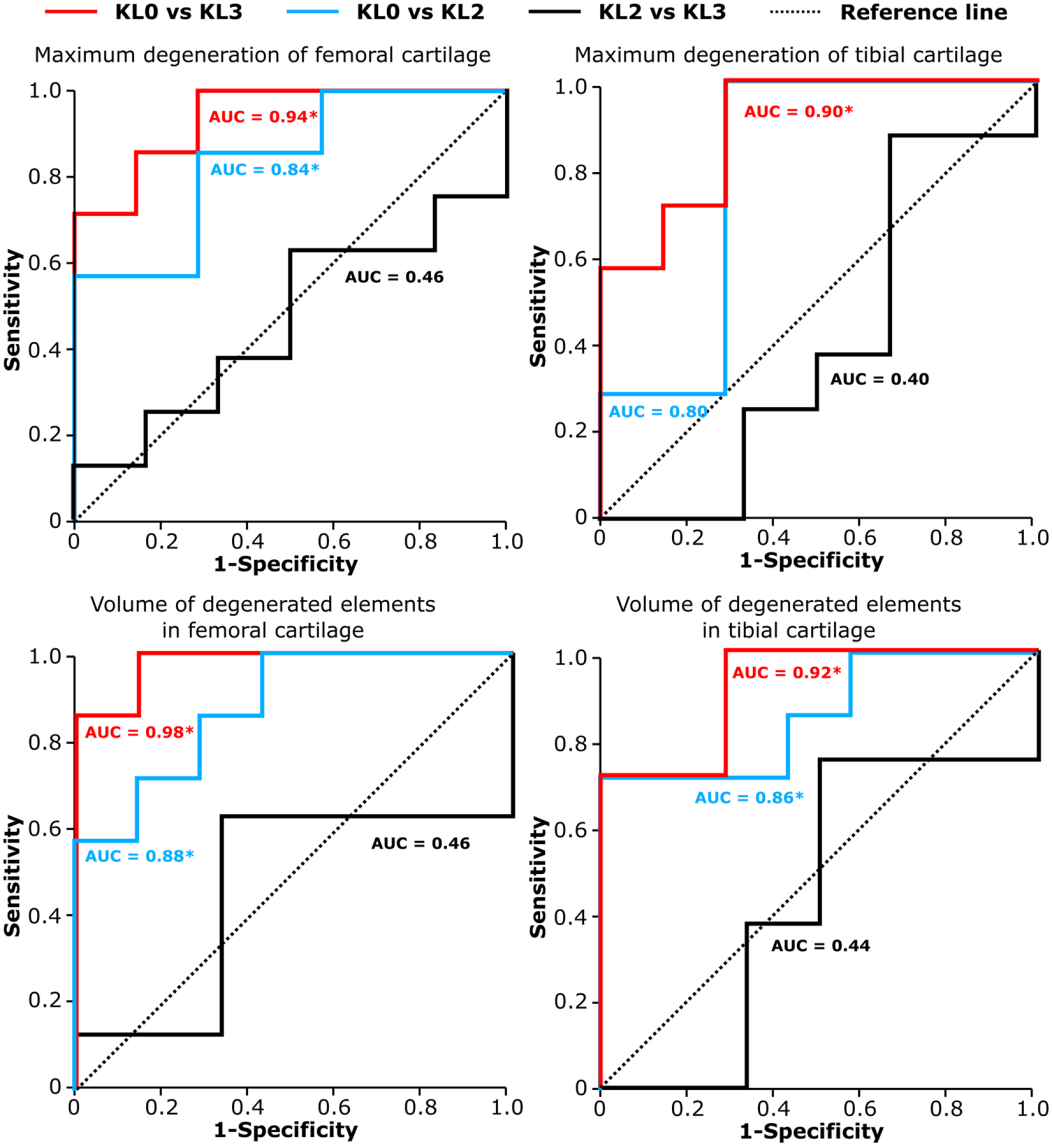
Receiving Operating Characteristics (ROC)-curves and Area Under Curve (AUC)–values (see statistical analysis in the Methods section) for the maximum degeneration and volume of degenerated elements of femoral and tibial cartilage. *p < 0.05.

Receiving Operating Characteristics (ROC) analysis confirmed that both the maximum degeneration and volume of degenerated elements after 100 iterations were significantly (*p* < 0.05) different between KL0 and KL3 group subjects as well as between KL0 and KL2 group subjects, except for the maximum degeneration in tibial cartilage between KL0 and KL2 groups (Fig. 6). The highest Area Under Curve (AUC) value was always obtained between KL0 and KL3 groups.

Matrices of classification errors (Table 1), calculated from the values after 100 iterations, showed that the level of OA was predicted adequately for KL0 and KL3 group subjects when the cut-off values were selected from the ROC curves such that the Youden index was maximized. Maximum degeneration of tibial cartilage predicted adequately also the level of OA for KL2 group subjects. On the other hand, the most confusion occurred in the prediction of the level of OA for KL2 group subjects when the maximum degeneration of femoral cartilage and volume of degenerated elements were studied.

**Table 1:**
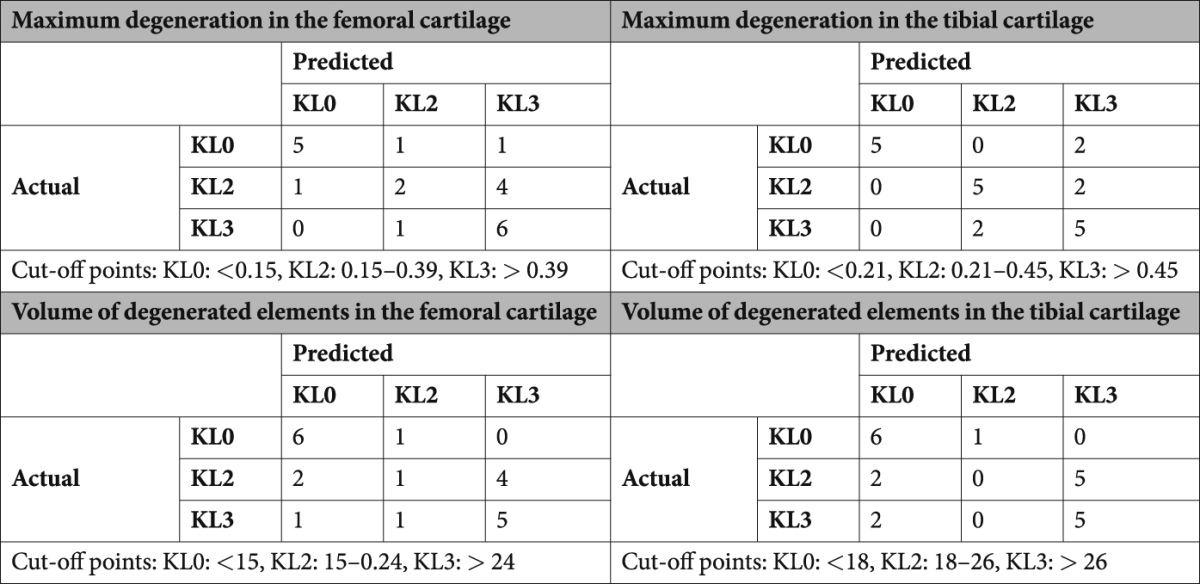
Matrices of classification errors for maximum degenerations and volumes of degenerated elements of femoral and tibial cartilage. Cut-off values for predicted KL grades were chosen from the ROC curves so that the Youden index was maximized (see statistical analysis from the Methods section).

## Discussion

Using quantitative cartilage degeneration algorithm, we were able to separate subjects with different levels of radiographical OA (KL2 and KL3 groups) from healthy subjects (KL0 group) after the 4-year follow-up period. It should be highlighted that the algorithm was even able to show higher degeneration levels for KL3 than KL2 group, even though these groups had the same BMI. These results highlight the potential of our degeneration algorithm to predict the subject-specific overloading-related progression of the knee OA.

The analysis of degenerated volumes within articular cartilage was able to separate both KL2 and KL3 subjects from KL0 subjects (Figs 5 and 6). Maximum degeneration parameter was also able to separate successfully KL2 and KL3 subjects from KL0 subjects in the femoral cartilage, but in the tibial cartilage, only KL3 subjects were separated statistically from KL0 group. These results suggest that the degenerated volume is likely more relevant predictive parameter since in more severe OA larger area of degenerated cartilage can be seen in the knee joint as verified in our subjects by MOAKS grading. In terms of KL grade, local cartilage defects in relative small areas do not necessarily show any degenerative changes in radiographic evaluation^35^. Therefore, local cartilage degenerations may be as severe even though the radiographic degree of OA (KL2 or KL3 group) would be different.

In the analysis of confusion matrix (Table 1), cut-off points were selected so that the Youden index was maximized^36, 37^. Using that selection, the levels of OA were predicted fairly well for KL0 and KL3 groups. Most of confusion was observed in the prediction of the level of OA for KL2 group subjects in all the other parameters except in the predicted maximum degeneration of tibial cartilage. Changing the cut-off point could improve our predictions also for the KL2 group. On the other hand, increasing the subject number could improve the ROC curves leading to improvements in our predictions as well.

Radiographic KL grading is the most commonly used OA classification method in clinics and OAI database provides this information for almost all subjects (other grades such as MOAKS are provided only for small subset of the subjects). For that reason, subjects were divided into different OA groups based on radiographic KL classification^11, 38^ after 4-year follow-up. This can also be considered as a limitation of the study since previous studies have shown that KL grades may vary between different observers^11, 39^. On the other hand, a significant correlation in KL grades between observers was seen earlier^11^ and reproducibility of the KL classification method was shown to be good when subjects with KL ≥ 2 were studied^39^.

KL grading method estimates the degree of OA mainly based on the amount of osteophytes and joint space narrowing and cannot reveal any changes in cartilage. Our degeneration algorithm predicts directly cartilage degeneration, not bony changes or cartilage loss. Cartilage thinning was indirectly taken into account in our algorithm as cartilage became softer due to OA progression, which actually leads to joint space narrowing during joint loading. On the other hand, when comparing our simulation results to tibial cartilage changes in MRI (MOAKS scoring performed by the musculoskeletal radiologist), our method predicted reasonable well actual degeneration locations in central regions of medial tibial cartilage (Fig. 3). In femoral cartilage, based on the MOAKS scores, cartilage degenerations occurred primarily on the anterior part of femoral cartilage, *i*.*e*., on the patellofemoral contact region. Since our model did not take this contact into account, MOAKS comparison of femoral cartilage was not relevant.

In the current study the stress threshold inducing cartilage degeneration was fixed (7 MPa) while it could be different for different subjects and it may vary during the progression of OA. Smaller threshold value in more degenerated cartilage would likely increase the area and volume of degenerated elements in the model and even improve the prediction of cartilage degeneration.

First signs of OA in cartilage tissue are collagen fibrillation, proteoglycan loss and increase in water content^40^. Since collagen controls strongly the mechanical response of cartilage under dynamic loading^41, 42^, such as walking which was simulated, and collagen network damage due to overloading can initiate OA^43, 44^, only collagen fibrillation was considered in our algorithm by decreasing the collagen network stiffness, while other factors (proteoglycan loss, water content) were not taken into account here. Those factors should be considered in the degeneration algorithm in the future to test if they would even improve the predictive value of the current algorithm. However, in order to validate such an improvement in the algorithm to separate degenerative changes in different tissue constituents, quantitative follow-up information (*e*.*g* some specific MRI parameters^45^) of specific changes in proteoglycan and water content during the progression of OA would be needed.

The subject-specific knee joint geometries were obtained from MRI, meaning that anatomical factors (shape and knee alignment) were taken into account in our degeneration model. On the other hand, knee loading was taken from the literature, since there was no subject-specific gait data in the OAI database. The individual gait data could improve the accuracy of our method and transfer degeneration locations even closer to actual changes observed in 4-year follow-up MRI (Fig. 3). Unlike MRI of the knee joint, nowadays in clinical practice collecting subject-specific gait data is not usually feasible, since measurements and data analysis are time consuming and gait analysis may not provide sufficient information for treatment planning. Therefore, it is encouraging that even without this data our predictions matched well with the radiographic and MRI evaluations, especially in the tibial cartilage.

Due to simplicity and initial estimation of the time needed for model generation and computations, quadriceps forces, patella, ligaments and muscles were neglected from the model. However, effects of patella as well as effects of muscles and ligaments were considered in external load in terms of total knee joint reaction forces between the tibiofemoral joint. This load input was also the same as used before^34^. It is known that knee OA can be seen also in patellofemoral joint, and thus, in such an analysis patella should be included in the model. However, it is also commonly known that OA develops primarily in the medial compartment^35, 46^ as was verified by our subjects’ MOAKS scores. Therefore, the analysis of only medial compartments was considered feasible in this study.

The total number of subjects used in this study was 21 divided into three different KL groups. This number is small but sufficient for statistical comparison. To our knowledge, this is the first whole knee joint FE modelling study, where the subject number is this large. In most of the previous knee joint finite element studies, the subject number was restricted to one or two^47–49^. On the other hand, since our inclusion criteria were tight, there were only 7 suitable subjects for KL3 group in OAI database. We wanted to keep our number of subjects in each group the same, thus in order to increase the subject number we should have loosened our inclusion criteria and include subjects whose OA may have been initiated due to other factors than overweight (for instance injury or surgery). However, in this study we wanted to keep groups homogeneous for a fair comparison. Furthermore, manual segmentation as well as creation and simulation of FE models for 21 subjects are time consuming, since there does not exist automatic phases during model generation. Naturally, higher subject number might statistically even improve our predictions for OA development and separate better KL2 from KL3 grade group. On the other hand, there were already statistically significant differences between healthy (KL0) and OA (KL2 and KL3) groups with the used subject number. Nonetheless, before the current method can be used as a clinical application in personalized healthcare, it should be tested and validated with higher subject number and with different subject groups.

Our degeneration algorithm is based on excessive and cumulative loads due to overweight, since it has been suggested in earlier studies that those are the risk factors for the onset and progression of OA^16^ and obesity is one of the major risk factors for knee OA^50^. On the other hand, it is known that there exist also other risk factors than load, such as gender^51, 52^, aging^51^, physical activity level^53, 54^ and metabolic changes (*e*.*g*. inflammation)^55, 56^. In the future, some of those factors could be taken into account in the degeneration algorithm. One option would be to adjust the threshold level for degeneration based on different risk factors. For instance, a previous study shows that cartilage failure limit is highly dependent on age of the subject^57^. Failure limit increases from ~25 to ~40 MPa until the age of 20–30 years and after that it starts to decrease to <10 MPa. On the other hand, estimation of subject-specific metabolic changes during the follow-up period is challenging, but if those could be reliable estimated and implemented into the algorithm, prediction of OA progression could be improved. However, first we would need to have solid statistical evidence how these parameters would affect KL or MOAKS gradings. It should be also noted that even the presented algorithm, purely based on mechanical overloading, was able to statistically separate healthy subjects from those with OA. It was also able to give higher degenerative levels for KL3 subjects than for KL2 subjects. Thus, at this point it is not possible to evaluate whether those additions to the algorithm would improve the prediction or not.

Since our goal was to predict local cartilage degeneration due to biomechanical overloading of the joint, a FE based method was applied. Therefore, data-driven methods were not used. One way to use the data-driven methods with our FE model and algorithm would be to add biomechanical factors into the statistical or machine learning based predictive models. However, in practice this would necessitate FE analysis of hundreds of subjects which would take years just to create those kinds of biomechanical models. In addition, statistical or machine learning methods would approach a template-based analysis, meaning that there would be lack of subject-specificity in terms of knee geometry and mechanics. This would then change the purpose of our current study.

It is estimated that annual costs per patient are increasing as Kellgren-Lawrence grade is increasing^58^ and costs for end-stage OA (prior to hip or knee replacement) are average of $3800 per person^59^. It is also suggested that the amount of total knee replacements will increase more than 600% by 2030 resulting in ~3.5 million operations in the US^60^. Our degeneration algorithm was able to predict cartilage degeneration for obese subjects and separate groups with different levels of OA. Thus, it provides a novel tool to predict quantitatively the subject-specific levels of cartilage degenerations and estimate progression of OA. By using this approach, effects of different interventions, such as weight loss, rehabilitation, and surgery, on OA progression could be simulated in a personalized medicine manner. Therefore, this algorithm could be applied as a clinical tool to improve personalized OA treatment planning by “optimizing” the intervention. This could decelerate or prevent the progression of OA and delay the need for total knee replacement surgery. Thus, it could help in economical savings for societies and obtain better quality of life, especially for elderly.

## Methods

### Osteoarthritis Initiative subjects

OAI datasets AllClinical00 and AllClinical06 were used for BMI, age, health status of menisci, and previous knee injuries. Datasets 0.C.2 and 0.E.1 were used for baseline MRI and 6.C.1 and 6.E.1 for 4-year follow-up MRI, whereas kxr_sq_bu00 and kxr_sq_bu06 were used for baseline and follow-up Kellgren-Lawrence grades, respectively. Ethical approval for collecting all subject information was provided by the OAI. Knee MRI’s were carried out in accordance with FDA guidelines, whereas knee radiographs (x-ray) were carried out in accordance with typical guidelines for annual and total radiation dosage to research subjects. Informed consent was obtained from all subjects prior to each clinic visit. The committee on human research is the institutional review board for the University of California, San Francisco (UCSF), and its affiliates. Since data collection was performed in many places, possible changes in OAI study protocol were reviewed and approved by local institutional review boards. Further details related to the OAI data are available in the OAI web-site (http://www.oai.ucsf.edu/).

### Magnetic Resonance Imaging Osteoarthritis Knee Score (MOAKS) –grading

The MR images were obtained with clinical 3 T MRI device (Siemens, Erlangen, Germany) using a dual-echo steady-state imaging sequence (SAG 3D DESS, TR = 16.32 ms, TE = 4.71 ms, flip angle = 25°, voxel size = 0.36 × 0.36 × 0.7 mm). The severity of structural changes was graded using a simplified MRI OA Knee Score (MOAKS) in separate knee locations^61^. In this study, we were focusing only on changes in articular cartilage and especially in tibiofemoral contact region, which is a part in the original MOAKS grading system. In MOAKS, we analyzed sizes of articular cartilage losses such that 0: no cartilage loss, 1: cartilage loss is < 10%, 2: cartilage loss is 10–75%, and 3: cartilage loss is > 75% of region of cartilage surface area. Femoral and tibial cartilages were divided into three different regions (anterior, central and posterior), but in femoral cartilage only central region being in contact with tibial cartilage during walking was analyzed. This is justified by the applied joint motion (joint forces concentrated mainly in central region in femoral cartilage) and our method does not require information about the forces in patellofemoral contact region. A musculoskeletal radiologist performed the MOAKS grading and he was unaware of subject’s characteristics, clinical and radiographic data.

### Geometry creation and finite element (FE) models

Knee joint geometries and finite element models were generated similarly as in a previous study^34^. MRI sequence used for segmentation was the same as described in MOAKS grading section. Tibial and femoral cartilages and menisci were segmented manually from the MR images using Matlab v7.14 (Mathworks Inc., Natick, MA, USA) (Fig. 2A). 3D knee joint geometries were created using Mimics v15.01 (Materialise, Leuven, Belgium) and final geometries were imported to Abaqus modelling software (Abaqus v6.13-3, Dassault Systèmes, Providence, RI, USA), where finite element models were constructed.

Cartilages were meshed using first-order, 8-node porous continuum hexahedral elements (element type = C3D8P) and first-order, 8-node continuum hexahedral elements without porosity (element type = C3D8) were used for meshing menisci. Element sizes in the whole knee joint model were ~1.5 mm, ~0.7 mm and ~1 mm for femoral and tibial cartilage and menisci, respectively. To investigate cartilage degeneration, medial compartment models were created (Fig. 2B). In those models, element sizes were ~1 mm and ~0.5 mm in femoral and tibial cartilage, respectively.

Fibril-reinforced poroviscoelastic (FRPVE) material was implemented for cartilages and transversely isotropic elastic (TI) material for menisci (Table 2). In the FRPVE material model, cartilage is composed of nonfibrillar and fibrillar matrices, in which the nonfibrillar part describes proteoglycans and interstitial fluid and the fibrillar part describes collagens. The fibrillar matrix consists of well-oriented primary and randomly-oriented secondary collagen fibrils^62^. The total stress ***σ***_tot_ is the sum of the solid nonfibrillar matrix stress ***σ***_nf_, fibrillar matrix stress (sum of primary and secondary fibril stresses) ***σ***_f_, and fluid pressure *p*:

1 $$\,{{\boldsymbol{\sigma }}}_{{\rm{tot}}}={{\boldsymbol{\sigma }}}_{{\rm{nf}}}+{{\boldsymbol{\sigma }}}_{{\rm{f}}}+p{\boldsymbol{I}},$$

where ***I*** is the unit tensor. Detailed description of the FRPVE material and implementation of the FRPVE and TI materials into the FE models can be seen from previous studies^34, 62^.

**Table 2:** Material parameters for cartilage^19^ and menisci^65, 67–69^.

Parameter	Tibial cartilage	Femoral cartilage	Menisci
**E**_**m**_ (**MPa**)	0.106	0.215	
**E**_**0**_ (**MPa**)	0.18	0.92	
**E**_*ε*_ (**MPa**)	23.6	150	
**ν**_**m**_	0.15	0.15	
**η** (**MPas**)	1062	1062	
**k**_**0**_ (**x 10** ^**−15**^ **m**)^**4**^ **/Ns**	18	6	
**n**_**f**_	0.8–0.15z	0.8–0.15z	
**E**_**1**_, **E**_**2**_ (**MPa**)			20
**E**_**3**_ (**MPa**)			159.6
**ν**_**12**_			0.3
**ν**_**31**_			0.78
**G**_**13**_ (**MPa**)			50

E_m = _nonfibrillar modulus, E_0_ = initial fibril network modulus, E_ε_ **= **strain-dependent fibril network modulus, ν_m = _Poisson’s ratio, η = viscoelastic damping coefficient, k_0 = _permeability, n_f = _fluid fraction, E_1_, E_2_, E_3_ = radial, axial and circumferential Young’s moduli, respectively, ν_12_, ν_31 = _Poisson’s ratios, G_13 = _shear modulus.

Since OAI database does not have subject-specific gait data, generic gait cycle from previous experimental studies^63, 64^ was implemented into the FE models, similarly as in a previous study^34^ (Fig. 2A). Gait cycle was implemented by three separate steps as follows: 1) Prior to the gait load, cartilages were first brought into a light contact; 2) Subsequently, initial axial load and flexion angle from the experiments were implemented into the model; 3) Finally, entire gait cycle was implemented and it was controlled by using time-dependent motion data (scaled to the subject’s body weight). Total duration of the stance phase of gait was 0.8 s. More detailed description of load implementation, boundary conditions and contact definitions can be seen from a previous study^34^.

The medial compartment models without menisci for the degeneration simulations was created^34^ to improve the computational efficiency and convergence (Fig. 2B). Time-dependent supportive effect of medial menisci was calculated and reduced from axial load extracted from the simulations of the whole knee joint model. In addition, flexion-extension angle and varus-valgus rotation were extracted from the whole knee joint models and extracted tibio-femoral loads and rotations were implemented into the medial compartment models (Fig. 2B). Finally, the iterative cartilage degeneration algorithm was included and the computational models were simulated using 100 iterative degenerative steps to get simulation results in a reasonable time (Fig. 2C).

### Cartilage degeneration algorithm

Since excessive loading and accumulated loads have been suggested to cause mechanical failure in cartilage^16^, our degeneration algorithm was based not only on impact loading but also duration of the load in different regions of the cartilage during walking. Only collagen was allowed to degrade in the algorithm, since collagen fibrillation is one of the first signs of OA reducing collagen matrix stiffness and controlling mechanical response of the cartilage during dynamic loads such as walking^40–42^. In the algorithm, local collagen fibril degeneration occurs if tensile stress of 7 MPa (cartilage failure limit^34, 65^) is exceeded at any time point during walking^34^. Fibril degeneration was controlled iteratively by reducing the collagen fibril network stiffness of the FRPVE material.

Cartilage fibril degeneration algorithm worked as follows: The current level of collagen fibril degeneration *D*_*i*_ in each element was considered as a weighted mean after each *i*:th iteration (gait cycle) so that

2 $$\begin{array}{ll}{D}_{i}=\,{D}_{i-1}-({D}_{i-1}\,\times \,\sqrt[1.5]{\sum _{t=1}^{TOT}{D}_{t}\times IN{C}_{t}}), & \mathrm{for}\,i > 1\\ {D}_{i}=1, & {\rm{for}}\,i=1\end{array}$$

where *D*_*i*−1_ is the degree of fibril degeneration in each element in the previous gait simulation, *TOT* is the total number of time increments in each simulation and *INC*_*t*_ is the duration of time increment *t*. *D*_*t*_ is the fibril degeneration factor for an individual element after each time increment and it describes the amount of fibril degeneration as a function of time. Fibril degeneration was assumed to occur if a collagen failure threshold *T* (7 MPa) is exceeded. Factor *D*_t_ was calculated as follows:

3 $$\begin{array}{ll}{D}_{t}=\,(\frac{({S}_{t}-T)/T}{100}), & {\rm{for}}\,{S}_{t} > T\\ {D}_{t}=0, & {\rm{for}}\,{S}_{t}\le T\end{array}$$

where *S*_*t*_ is the local tensile stress during each time increment. *D*_t_ = 0 means no degeneration and *D*_t_ = 1 means fully degenerated collagen fibrils. Finally, calculated fibril degeneration *D*_i_ was implemented into the FE model by modulating the total fibril stress tensor ***σ***_f_ from equation (1):

4 $${{\boldsymbol{\sigma }}}_{{\rm{f}}}=\,\sum _{j=1}^{totf}{{\boldsymbol{\sigma }}}_{f,j}{\bar{e}}_{f,j}\otimes {\bar{e}}_{f,j},$$

where *totf* is the total number of fibrils (secondary and primary), ${\bar{e}}_{f,j}$ is the fibril direction and *σ*_f,j_ are the stresses of primary and secondary fibrils:

5 $$\begin{array}{c}{\sigma }_{f,j}=\rho {D}_{i}C{\sigma }_{f}\,({\rm{for}}\,{\rm{primary}}\,{\rm{fibrils}}),\\ {\sigma }_{f,j}=\rho {D}_{i}{\sigma }_{f}\,({\rm{for}}\,{\rm{secondary}}\,{\rm{fibrils}}),\end{array}$$

where ρ is the fibril density, C is a fraction between primary and secondary fibrils and *σ*_f_ is the viscoelastic fibril stress^62^:

6 $$\begin{array}{ll}{{\sigma }}_{f}\frac{\eta }{2\sqrt{{\sigma }_{f}-{E}_{0}{\varepsilon }_{f}}{E}_{\varepsilon }}{\dot{\sigma }}_{f}+{E}_{0}{\varepsilon }_{f}+(\eta +\frac{\eta {E}_{0}}{\sqrt{{\sigma }_{f}{E}_{0}{\varepsilon }_{f}}{E}_{\varepsilon }}){\dot{\varepsilon }}_{f}, & {\rm{if}}\,{\varepsilon }_{f} > 0\\ {\sigma }_{f}=0, & {\rm{if}}\,{\varepsilon }_{f}\le 0\end{array}$$

where *η* is a damping coefficient, *E*_0_ and *E*_*ε*_ are initial and strain-dependent fibril network moduli, respectively, *ε*_*f*_ is the fibril strain, and ${\dot{\sigma }}_{f}\,{\rm{and}}\,{\dot{\varepsilon }}_{f}$ are stress- and strain rates, respectively.

Finally, above described procedure is repeated for all elements of the FE model and after that new iteration (gait cycle) is started.

### Statistical analysis

Mann-Whitney U-test was used for statistical comparison between different KL-groups in volumes of degenerated elements and maximum degeneration levels of the medial tibial and femoral cartilages. Predictive accuracy of the algorithm was tested using Receiving Operating Characteristics (ROC) and Area Under Curve (AUC)^66^. In addition, matrices of classification errors were calculated using the Youden index criteria for cut-off values, i.e. the vertical distance from the reference line to the cut-off point was maximized^36, 37^. The level of significance was set to *p < *0.05. Statistical analysis was performed using SPSS software (SPSS 23.0, SPSS Inc., Chicago, IL, USA).

### Data availability statement

The data that support the findings of this study are available from the corresponding authors upon reasonable request.
